# Spectral Analysis of Human Retinal Pigment Epithelium Cells in Healthy and AMD Eyes

**DOI:** 10.1167/iovs.65.1.10

**Published:** 2024-01-03

**Authors:** Leonie Bourauel, Marc Vaisband, Leon von der Emde, Katharina Bermond, Ioana Sandra Tarau, Rainer Heintzmann, Frank G. Holz, Christine A. Curcio, Jan Hasenauer, Thomas Ach

**Affiliations:** 1 Department of Ophthalmology, University of Bonn, Bonn, Germany; 2 Institute of Life & Medical Sciences, University of Bonn, Bonn, Germany; 3 Department of Internal Medicine III with Haematology, Medical Oncology, Haemostaseology, Infectiology and Rheumatology, Oncologic Center, Salzburg Cancer Research Institute Laboratory for Immunological and Molecular Cancer Research, Paracelsus Medical University, Salzburg, Austria; 4 Cancer Cluster Salzburg, Salzburg, Austria; 5 Department of Ophthalmology, Ludwigshafen Hospital, Ludwigshafen, Germany; 6 Department of Ophthalmology, Asklepios Klinik Nord - Heidberg, Hamburg, Germany; 7 Leibniz Institute of Photonic Technology, Jena, Germany; 8 Institute of Physical Chemistry and Abbe Center of Photonics, Friedrich-Schiller University Jena, Jena, Germany; 9 Department of Ophthalmology, University of Alabama at Birmingham, Alabama, Alabama, United States

**Keywords:** autofluorescence, lipofuscin, melanolipofuscin, emission spectrum, retinal pigment epithelium, confocal fluorescence microscopy

## Abstract

**Purpose:**

Retinal pigment epithelium (RPE) cells show strong autofluorescence (AF). Here, we characterize the AF spectra of individual RPE cells in healthy eyes and those affected by age-related macular degeneration (AMD) and investigate associations between AF spectral response and the number of intracellular AF granules per cell.

**Methods:**

RPE–Bruch's membrane flatmounts of 22 human donor eyes, including seven AMD-affected eyes (early AMD, three; geographic atrophy, one; neovascular, three) and 15 unaffected macula (<51 years, eight; >80 years, seven), were imaged at the fovea, perifovea, and near-periphery using confocal AF microscopy (excitation 488 nm), and emission spectra were recorded (500–710 nm). RPE cells were manually segmented with computer assistance and stratified by disease status, and emission spectra were analyzed using cubic spline transforms. Intracellular granules were manually counted and classified. Linear mixed models were used to investigate associations between spectra and the number of intracellular granules.

**Results:**

Spectra of 5549 RPE cells were recorded. The spectra of RPE cells in healthy eyes showed similar emission curves that peaked at 580 nm for fovea and perifovea and at 575 and 580 nm for near-periphery. RPE spectral curves in AMD eyes differed significantly, being blue shifted by 10 nm toward shorter wavelengths. No significant association coefficients were found between wavelengths and granule counts.

**Conclusions:**

This large series of RPE cell emission spectra at precisely predefined retinal locations showed a hypsochromic spectral shift in AMD. Combining different microscopy techniques, our work has identified cellular RPE spectral AF and subcellular granule properties that will inform future in vivo investigations using single-cell imaging.

The retinal pigment epithelium (RPE) is a monolayer of polygonal cells essential for outer retinal health.^1^ Phagocytosis of photoreceptor outer segments; transport of oxygen, nutrients, and metabolites; and maintenance of the visual cycle are among the essential functions of the RPE.^2^ The daily lysosomal degradation of ingested photoreceptor outer segments leads to the intracellular accumulation of lipofuscin in postmitotic RPE cells. This accumulation begins early in childhood and continues during adulthood. Interestingly, at advanced ages and during the course of retinal diseases, including age-related macular degeneration (AMD), RPE cells tend to lose lipofuscin granules.^3^ Furthermore, AMD-affected RPE cells are characterized as enlarged with more variable shapes and sizes compared to unaffected aged retinas.^4^

Every RPE cell contains a variety of organelles/granules with distinct autofluorescent properties relevant to clinical imaging: The majority of granules are lipofuscin and melanolipofuscin, which exhibit strong autofluorescence (AF) after excitation with blue light (488 nm). In contrast, melanosomes show light-absorbing characteristics when excited at 488 nm.^5^^–^^7^ Regarding AF, in the aforementioned granules, fluorophores are the underlying molecules, which are probably bisretinoids from the visual cycle, among others, but many have not yet been described. Each fluorophore has its characteristic excitation and emission wavelengths.

Previously, we characterized RPE cells by total AF and granule load per cell, granule phenotype, and the distribution of granules at different retinal locations as defined by Polyak^8^: fovea (within 0.4 ± 0.5 mm superior to the foveal center), perifovea (within 4.1 ± 0.2 mm superior to the foveal center), and near-periphery (within 8.6 ± 1.3 mm superior to the foveal center). In healthy eyes, we could classify nine different granule phenotypes based on AF properties.^6^ Lipofuscin was highest at the perifovea and increased with age, whereas melanolipofuscin was the abundant granule type at the fovea and showed no changes in quantity in retinas of older donors.^6^ This characteristic accumulation seems to be related to the photoreceptor distribution, as lipofuscin load is abundant in areas of high rod density, whereas at the cone-rich fovea melanolipofuscin was the predominant autofluorescent granule type.^6^ In AMD-affected eyes, however, RPE cells showed a higher variability in cell size, granule load, and shape. In these eyes, lipofuscin fraction was reduced at the fovea, perifovea, and near-periphery compared to healthy eyes,^9^ a finding recently confirmed in clinical quantitative fundus autofluorescence (qAF) imaging with loss of AF in intermediate AMD.^10^^,^^11^

Therefore, histologic findings can inform clinical blue-light AF imaging. This non-invasive technique can be used to monitor RPE health in an unaffected retina and to diagnose retinal diseases such as AMD by mirroring subcellular granule distribution.^3^^,^^12^ In addition to AF intensity (as in qAF), AF can also be characterized by its spectral properties. Over the past 20 years, studies have characterized spectra of the RPE and Bruch's membrane in healthy and diseased eyes. AF emission spectra revealed that AMD is associated with blue-shifted emission AF of the RPE, sub-RPE deposits, and Bruch's membrane when excited at short wavelengths (λ_exc_ = 364 nm, 430 nm, and 488 nm), but no differences were observed for λ_exc_ of 568 or 633 nm.^13^^,^^14^ The peak emission wavelength for 480- to 488-nm wavelength light, however, was reported differently. Using histologic cross-sections, Marmorstein et al.^13^ reported peak emission at 555 nm for λ_exc_ of 488 nm, whereas Schultz et al.^15^ reported a peak emission at 610 nm for λ_exc_ of 480 nm. A slight hypsochromic shift was seen for RPE in AMD-affected tissues compared to healthy aged ones.^13^^,^^15^ Fluorescence intensity of suspended RPE cells was notably higher between 530 and 580 nm in eyes with AMD (λ_exc_ = 488 nm) but was reduced between 600 and 650 nm compared to control samples.^16^

As mentioned, available spectral data so far are from cross-sections or suspended RPE cells, but it is unclear what emission spectra look like in en face RPE microscopy which would reflect clinical in vivo imaging settings much more appropriately.

To assess this, we used RPE flatmounts from healthy young and aged donors, as well as AMD-affected donors, for autofluorescence imaging and emission spectral analysis. Spectral profiles of individual RPE cells at precisely defined retinal locations will help to further distinguish between healthy and diseased cells and eyes and can add valuable information for the interpretation of clinical fundus AF, including spectral analysis.

## Methods

This study was approved by the institutional review boards of the University of Alabama at Birmingham, the Ethics Committees of the University of Würzburg, and the University of Bonn (#386/20). All protocols adhered to the tenets of the Declaration of Helsinki.

### Tissues

Twenty-two RPE/Bruch's membrane flatmounts from 22 Caucasian donors of both sexes were used for this study. Some of the tissues have been previously characterized and used for different autofluorescent microscopy studies.^17^ In the current study, 15 eyes showed no macular pathology (<51 years, eight; >81 years, seven), and seven eyes were classified as AMD (age > 81 years; three early, one late non-exudative, and three late exudative).^18^ Briefly, donor tissues were treated as follows: Globes were collected from Advancing Sight Network (Birmingham, AL, USA) within a mean of 4.2 hours after death, preserved by immersion in 4% paraformaldehyde/0.1-M phosphate buffer, and inspected under a dissection microscope equipped with trans- and epi-illumination to further classify healthy or AMD stage. Following this, the neuroretina and choriocapillaris were removed in a multistep preparation and imaging process to ensure preservation of the exact foveal position (for details, see Supplementary Fig. S1 in Ach et al.^17^). RPE flatmounts were then imaged at three predefined locations using the regional definitions by Polyak^8^: fovea (0.4 ± 0.5 mm centered on the fovea), perifovea (4.1 ± 0.2 mm superior to the foveal center), and near-periphery (8.6 ± 1.3 mm superior to the foveal center) (Supplementary Fig. S1). These regions were chosen for the distinctive content of overlying photoreceptors (local rod-to-cone ratios of 0, 17.5, and ∼25, respectively).^19^^–^^21^

### Imaging Protocol

RPE flatmounts were imaged using a Zeiss LSM 780 laser scanning confocal fluorescence microscope (ZEISS Group, Jena, Germany).^6^ The excitation wavelength was set to 488 nm, and emission spectra were recorded from 490 to 695 nm in 24 channels with an 8.9-nm spectral channel width. Images were captured using a 63× (numerical aperture: 1.40) plan apochromat oil immersion objective (scanning area set at 224.92 × 224.92 µm^2^). *Z*-stacks of the LSM images were acquired from RPE cell bodies from apical (first autofluorescent granules in focus) to basal (last autofluorescent granules out of focus) in 390-nm steps.

### Image and Spectra Analysis

**Figure 1.**
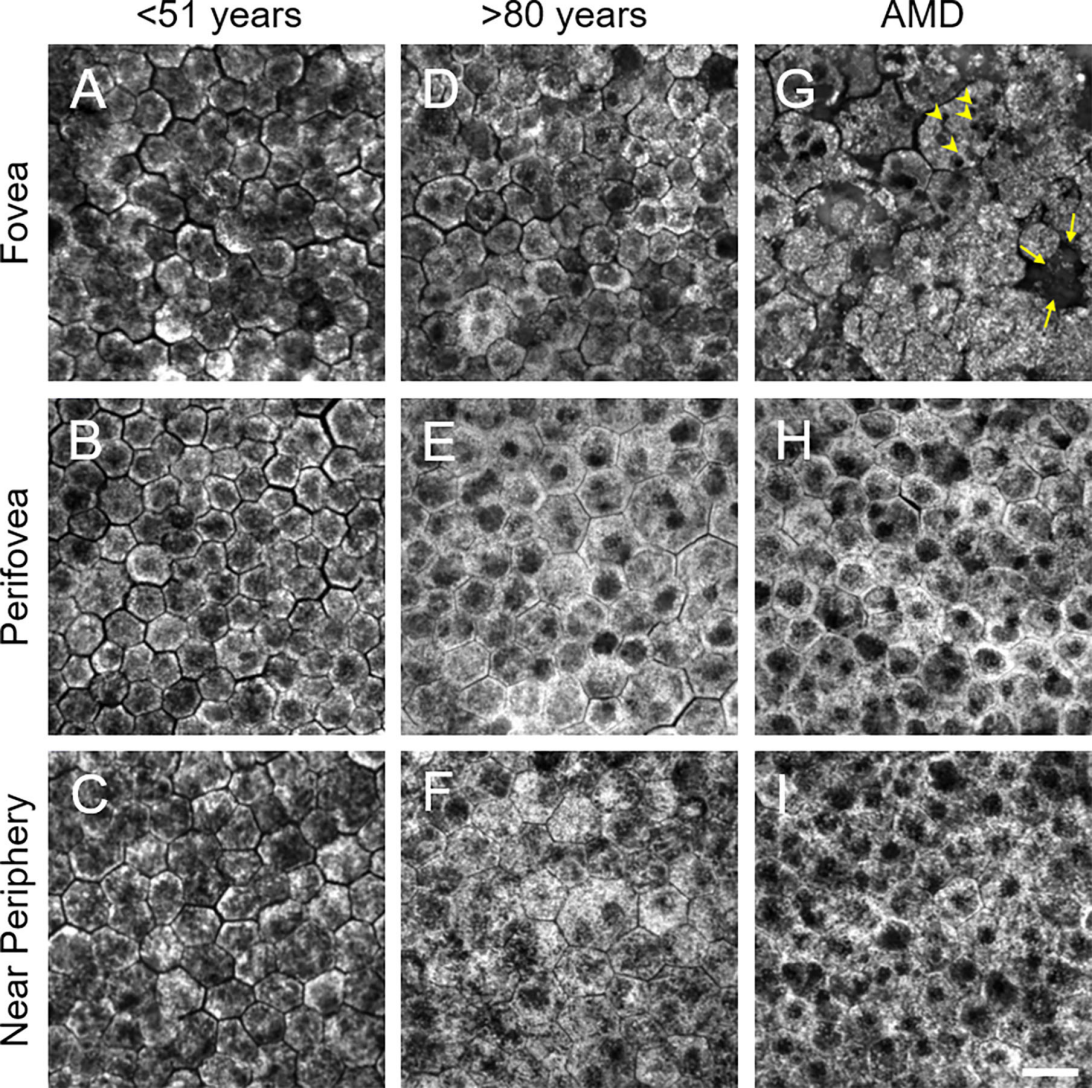
Representative RPE flatmount autofluorescence images for each age group and retinal location. For each group, *z*-projections of an RPE cell stack at the fovea (**A**, **D**, **G**), perifovea (**B**, **E**, **H**), and near-periphery (**C**, **F**, **I**) are shown. The fovea of AMD-affected tissues (**G**) shows multinucleated enlarged cells (*yellow arrowheads* point toward nuclei), non-distinguishable cell borders in the individual slabs of the *z*-projection, and not clearly visible cells due to loss or other reasons (*arrows* in **G**). The perifovea and near-periphery seem mostly unaffected compared to healthy tissues. Of note, RPE cells at the perifovea or near-periphery show not so clear cell borders. This, however, is not related to AMD but rather due to image processing (*z*-projection), as borders were visible in the individual slabs (data not shown). (**A**) Healthy 50-year-old female, (**B**) 36-year-old male, (**C**) 47-year-old female, (**D**, **F**) healthy 83-year-old female, (**E**) 90-year-old female, and (**G**–**I**) 81-year-old male with AMD. *Scale bar*: 20 µm.

For every tissue at each location (fovea, perifovea, and near-periphery) (Fig. 1), 100 to 150 adjacent RPE cells were analyzed (for exceptions, see below). Individual RPE cells were selected, and their cell boundaries (presumed plasma membranes) were marked using ImageJ Fiji (National Institutes of Health, Bethesda, MD, USA)^22^ as linear hypoautofluorescent gaps between two adjacent cells, visible while *z*-scanning through the RPE cell monolayer.^23^

At locations severely affected by AMD, adjacent cells were not always detectable due to loss of well-defined cell boundaries and the presence of multinucleate and giant RPE cells.^3^^,^^17^ Especially for the fovea, only a few RPE cells could be selected in some cases. No RPE was left at the fovea in three eyes with late exudative AMD, and subsequently no confocal images could be captured.

**Figure 2.**
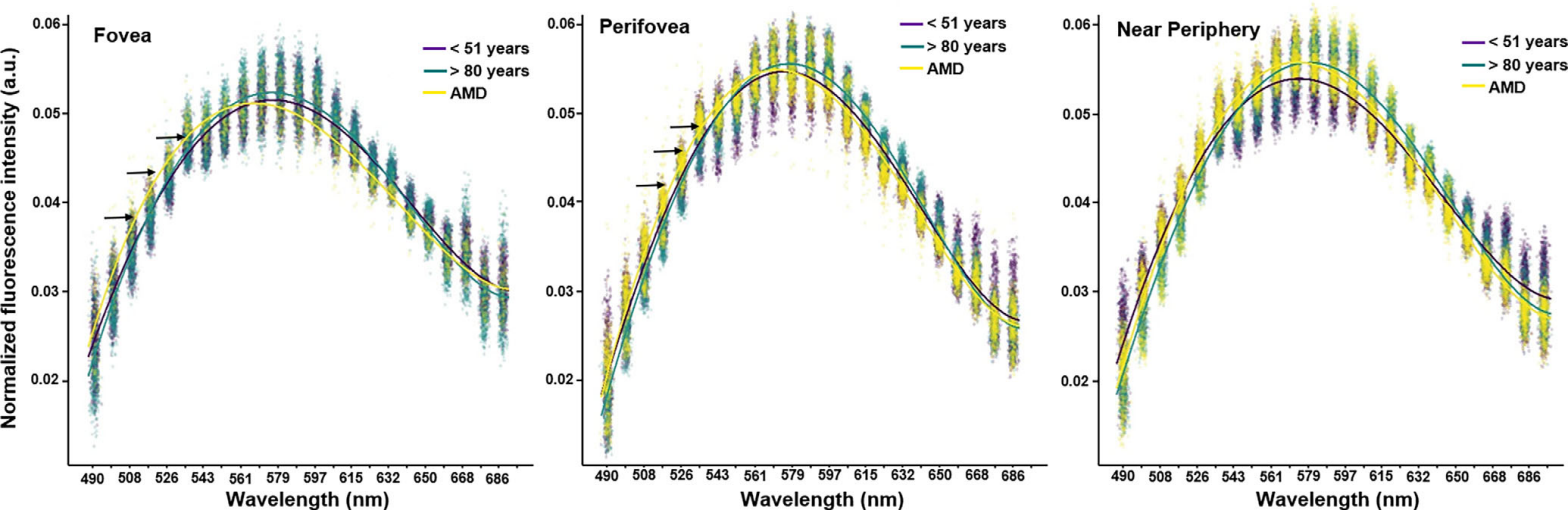
Scatterplots of averaged normalized emission spectra of RPE cells in healthy young (*purple*), healthy aged (*green*), and AMD (*yellow*) donors. Spectra are shown for the fovea, perifovea, and near-periphery, with least-square cubic splines fitted to all data from a group for visualization (the fitted splines serve only as visual aids and were not used for statistical analysis). A slight spectral shift toward shorter wavelengths for AMD spectra can be seen at the fovea and at the perifovea (*black arrows*).

Each cell was encircled within the LSM images using the computer-assisted Polygon Selection Tool in Fiji.^22^ Then, AF spectra were extracted from the image data using publicly available Fiji plugins.^24^^–^^26^ Single-cell spectral data recording, compilation, and integral normalization were carried out by using ImageJ plugins that were developed by Jay Unruh at the Stowers Institute (Kansas City, MO, USA); they are available on their website or via the ImageJ updater (Supplementary Table S1). Analysis was carried out as follows: Marked cells were selected one after another, and a spectrum for every single cell was automatically generated with the Fiji plugin “create spectrum jru v1.” Spectra of single cells were automatically grouped per donor and location using the “combine all trajectories jru v1” Fiji plugin. For comparability, integral normalization was performed on all fluorescence spectra (“normalize trajectories jru v1” plugin). Spectral data for every channel were registered and exported to a text file for further statistical analysis. The source codes for all applied plugins are available via the Fiji/ImageJ updater (https://research.stowers.org/imagejplugins/updates/) or via the developer's website (https://research.stowers.org/imagejplugins/zipped_plugins.html). For further analysis and to facilitate visualization, mean spectra per group or donor and localization were generated (see Figs. 2 and 5). Spectral data are reported for wavelengths from 500 to 698 nm. Data for far-red light (>700 nm) were not used for analysis because optics of the microscope were not optimized for wavelengths >700 nm.

### Intracellular Lipofuscin and Melanolipofuscin Granule Load

Ten cells per location were further analyzed to correlate RPE cell AF spectra with the granule load of the RPE cells, using granule data from previous work.^6^^,^^9^ In brief, intracellular granules were manually tagged in super-resolution structured illumination microscopy RPE *Z*-stacks and classified on the basis of granule morphology and AF properties. We focused on lipofuscin, melanolipofuscin, and melanosomes (Table 1).^6^^,^^27^^,^^28^ The number of granules per cell was then correlated with the spectrum of the cell (see Statistical Analysis).

**Table 1.** Number of Granules per RPE Cell in Healthy Young (<51 Years), Healthy Old (>80 Years), and AMD-Affected Donor Eyes

		Location, Mean ± SD		
Granules	Eyes	Fovea, *n*	Perifovea, *n*	Near-Periphery, *n*
Lipofuscin	<51 y	74 ± 61	296 ± 182	189 ± 112
	>80 y	106 ± 78	318 ± 152	293 ± 261
	AMD	111 ± 58	580 ± 270	418 ± 208
Melanolipofuscin	<51 y	224 ± 56	210 ± 86	202 ± 66
	>80 y	228 ± 84	189 ± 76	226 ± 89
	AMD	497 ± 278	486 ± 302	535 ± 229
Melanosomes	<51 y	10 ± 17	4 ± 7	8 ± 16
	>80 y	4 ± 5	2 ± 3	2 ± 3
	AMD	5 ± 6	3 ± 3	3 ± 4

The higher granule numbers in AMD cells can be explained by cell enlargement and, therefore, higher cell volume (data not shown).^9^

### Total Autofluorescence Per Cell

To determine the total autofluorescence for each cell, *Z*-stacks were sum-projected so the intensities of every pixel of all optical slices were added pixelwise over the *z*-direction. In a second step, intensities of all pixels within this two-dimensional image were summed, which resulted in the total AF intensity of the sum-projected cell. Autofluorescence imaging procedures and camera settings were identical between donors and retinal location; however, no reference for AF quantification was used during microscopy.

### Statistical Analysis

To obtain a qualitative overview of the normalized emission spectra, we investigated the typical peak location by calculating for each cell the wavelength at which the spectrum was maximal. To obtain the spectrum peak for a certain location (fovea, perifovea, or near-periphery) per eye, we then considered the median over all cells and recorded the median peak location for each combination of donor group and location.

Regarding the differences in the number of available cells for analysis across disease stages, we did not choose to consider all examined cells as separate but potentially dependent data points. Instead, we opted for the statistically conservative approach of utilizing the eye-wide median to obtain one independent data point per eye.

For quantitative statistical analysis, dimensionality reduction was performed. To this end, we utilized principal component analysis (PCA) on the emission intensities.^29^ This is a linear dimensionality reduction method that represents the input data in an orthonormalized coordinate system whose axes (the eponymous *principal components*) are chosen to maximize the observed variance. By keeping only a number of PCs, in order of most explained variance, input data can be represented in terms of fewer compound summary statistics, which are themselves weighted sums of the input features.

The aforementioned integral normalization enforced that the sum over all channels was 1 for each data point. As a consequence, all input features were on comparable scales, so that no additional data transformations were performed before PCA. Applying PCA then reduced the data dimensionality from 24 (the number of emission spectra channels used; 8.9-nm steps) to 2. To provide a geometrical interpretation of the resulting features, we visualized the principal components (PCs; also referred to as loadings) by plotting the median normalized spectrum and superimposing the results of perturbing it in the direction of PC1 or PC2.

With the PCA-transformed data as the final condensed features, we performed several analyses. First, we used nonparametric tests to investigate differences between groups of cells. For this purpose, we considered the median PC1 and PC2 values per eye and used the Mann–Whitney *U* test to quantify differences between AMD cells and healthy controls. To examine differences between retinal locations, we did the same with a Kruskal–Wallis test, considering only eyes from donors in the healthy cohort.

In addition, we investigated the association between geometrical features of emission spectra, as quantified by the PCs, and the presence of granules in cells. Because the number of melanosomes in cells is variable in wholemounts due to preparation-related loss of apical processes, we used the data on lipofuscin and melanolipofuscin only.^6^

To correctly account for both intra- and interpatient variability and use the hierarchical structure of the data (cells nested in donor eyes), we employed linear mixed-effect models. For each PC and each type of granule, we fitted a linear mixed-effect model (as implemented by the lmerTest package for R^30^^,^^31^) as follows: The PCs and granule numbers were normalized to mean 0 and standard deviation 1. For each principal component and each granule type, a model was set up with the normalized PC as the exogenous variable and the normalized granule number as the endogenous variable. The random effects were chosen to be localization and donor identity, incorporating per-group intercepts into the model to account for the autocorrelation between cells of the same patient and from the same localization. The resulting non-intercept coefficients (± standard error estimate), although by necessity not correlation coefficients, can be interpreted on the same scale and represent a measure of association between two variables that accounts for our data structure. To detect differences in this relationship between groups, we additionally used a *z*-test to compare the resulting coefficients.^32^ A value of *P <* 0.05, with respect to the null hypothesis that the true association coefficient was zero, was considered statistically significant.

## Results

In this study, 22 RPE flatmounts from 15 healthy donor eyes (eight donors ≤ 51 years with mean age ± SD of 41.0 ± 11.5 years; seven donors > 80 years with mean age ± SD of 85.0 ± 3.0 years) and seven donors with AMD (85.0 ± 3.0 years; range, 81–90 years) were included. One healthy aged donor was excluded due to reduced image quality. In addition, spectra of foveal RPE cells in late neovascular AMD eyes were not available due to foveal RPE cell loss. Nonetheless, emission spectra of a total of 5.549 RPE cells from all three locations were analyzed (Table 2). Morphologically, healthy RPE cells showed a typical polygonal pattern. Increased cell area could be observed in healthy aged tissues (Fig. 1). For AMD, multinucleated RPE cells,^33^ loss of the hexagonal cell shape, and cell loss were apparent, especially at the fovea. Furthermore, foveal AF appeared to be reduced compared to that of healthy aged tissues (Fig. 1G vs. 1D).

**Table 2.** Number of Cells Analyzed

	Location, *n*		
Eyes	Fovea	Perifovea	Near-Periphery
<51 y	760	817	776
>80 y	652	681	719
AMD	92	475	570

### Spectra of RPE Cells

The mean spectral curves of the three groups (healthy young, healthy aged, AMD) showed a similar curve, with increasing AF emission from 490 nm onward with a peak at 570 to 580 nm at all locations (Fig. 2). Spectral peaks were very close in a 10-nm range dependent on group and location (Table 3). In AMD, the peak was around 570 nm in contrast to healthy young and healthy aged eyes (580 nm).

Figure 2 depicts a great variance in AF peak emissions for individual cells within the groups (see clusters of points around the curves). Variance in AF peak emission was more pronounced for individual donors than groups (age, AMD).

**Table 3.** Median Spectral Peaks for Each Group and Retinal Location

Eyes	Location, Median ± SD		
	Fovea (nm)	Perifovea (nm)	Near-Periphery (nm)
<51 y	578 ± 8.25	579 ± 7.71	575 ± 4.45
>80 y	582 ± 11.10	582 ± 6.63	578 ± 3.32
AMD	570 ± 0.00	572 ± 3.32	571 ± 5.69

For all cells, the wavelength with the highest peak was determined, and then, for each eye, the median highest peak wavelength to obtain a peak for the eye was taken. Finally, for all eyes of a group, we report the median. There appears to be a slight shift toward shorter wavelengths between healthy and AMD donors.

### Dimensionality Reduction and Quantitative Analysis of RPE Spectra

At the fovea, RPE cells of healthy young and aged donors had nearly identical AF emission spectral curves, with only a minor increase in spectral peak for healthy aged cells. In contrast, AMD-affected cells showed a blue shift of the whole spectrum at the fovea and at the perifovea. However, the differences in spectral shape or peaks between healthy eyes and AMD-affected eyes at the near-periphery cannot be interpreted unambiguously (Fig. 2, Table 3). A hypsochromic shift in AMD cells compared to healthy aged cells was apparent, but there was a difference of only 5 nm between AMD cells compared to healthy young cells.

**Figure 3.**
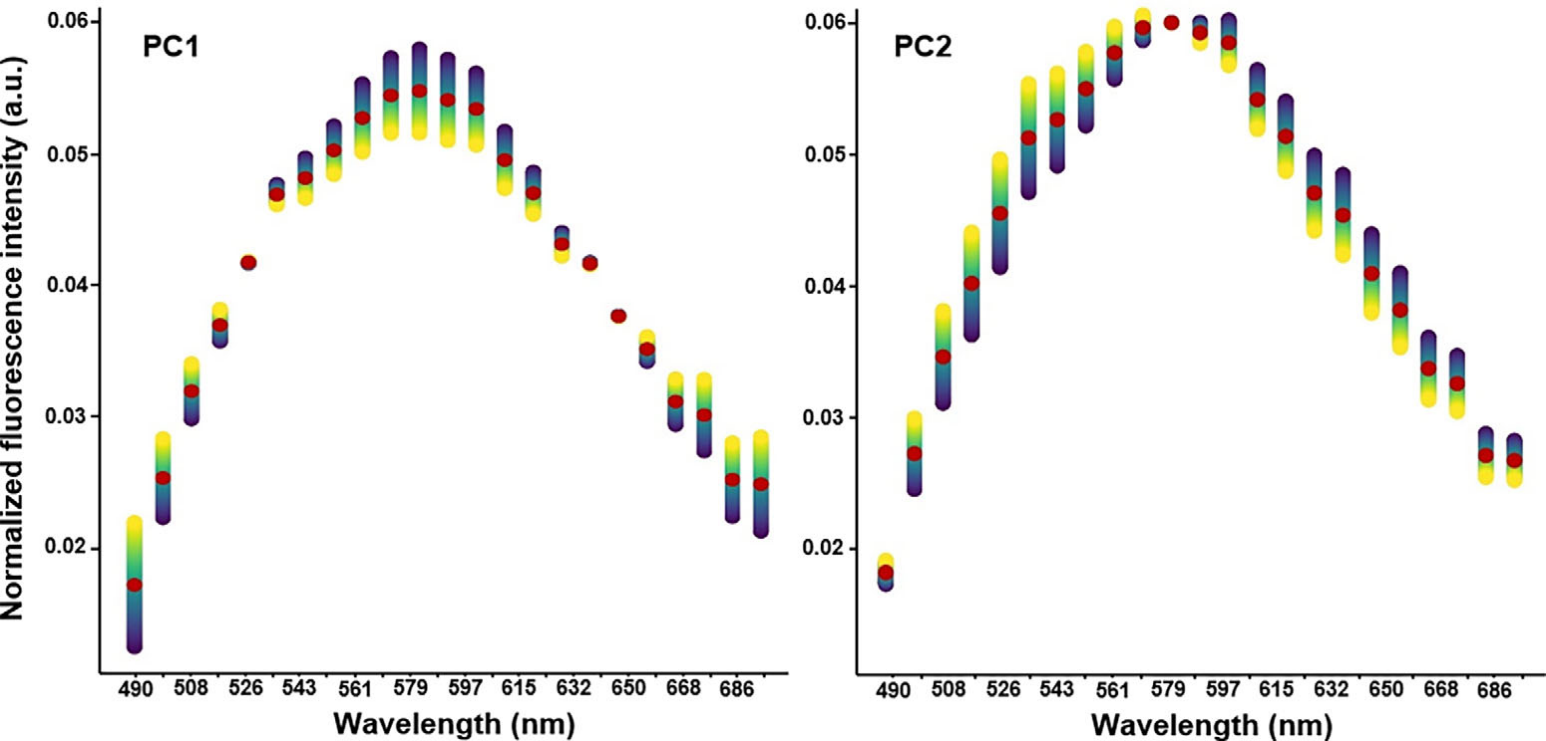
Visualization of principal components. To provide an interpretation for the two PCs, the figure shows a median normalized spectrum in *red*, which is then perturbed along the PCs (also referred to as “loadings” of the PCA). As such, the figure shows changes when a PC is increased (in *yellow*) or decreased (in *blue*). An increase in PC1 corresponds to a flattening of the emission curve, and an increase in PC2 is equivalent to a hypsochromic shift.

**Figure 4.**
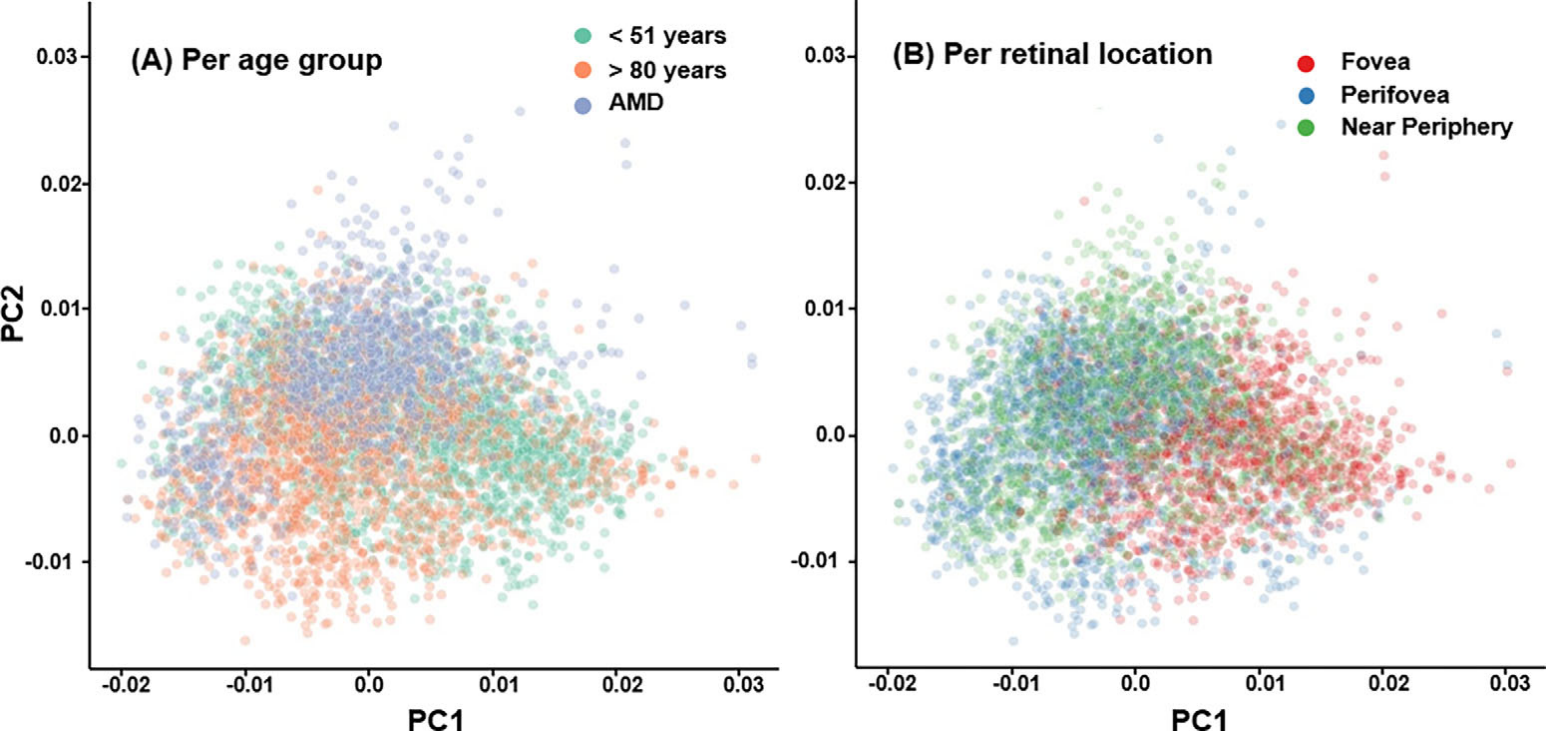
PCA of normalized emission spectra. Each *dot* represents a cell, with localization in the plot determined by PC1 and PC2. (**A**) Visualization by diagnostic group (young, aged, AMD) showing the differences between healthy young (<51 years) and aged donors (>80 years) with unaffected macula by PC1 and between healthy aged (>80 years) with unaffected macula and AMD by PC2. (**B**) Visualization by retinal location showing more PC1 in foveal cells, which should result in a flatter curve.

The PCA reduced the normalized spectra to two main characteristics: principal component 1 (PC1) and principal component 2 (PC2). The first two principal components explained 75.8% and 23.2% of the observed variance, respectively, yielding a cumulative variance ratio of 99.0% (Supplementary Fig. S2). This leads to the conclusion that two PCs provide an excellent reduced representation of the input data. A visualization of a median spectrum perturbed in the direction of the PCs appears to show that data points with high PC1 values represent curves that are flatter (i.e., have a smaller difference between peak and minimum intensity). PC2, meanwhile, appears tied to the position of the peak, with a high PC2 value being associated with a blue shift and shorter wavelengths (see Fig. 3). The results of the Mann–Whitney tests suggest that AMD tissues exhibit a higher PC2 value than that of healthy aged donors (fovea, *P* = 0.018; perifovea, *P* = 0.051; near-periphery, *P* = 0.128), whereas no significant differences in PC1 were observed (fovea, *P* = 0.192; perifovea, *P* = 0.274; near-periphery, *P* = 0.585) (for a PCA plot by group, see Fig. 4^5^A). This indicates a hypsochromic shift for AMD compared to healthy aged donors.

**Figure 5.**
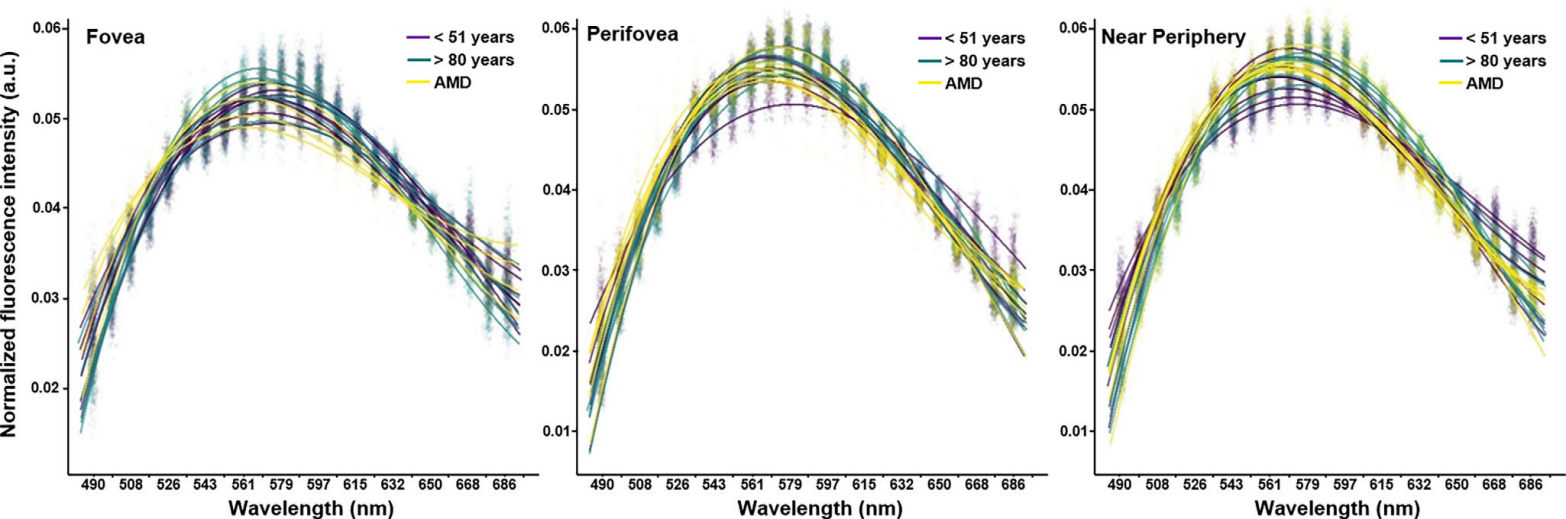
Averaged normalized emission spectra per donor of RPE cells. All analyzed cells are shown as scatterplots, overlaid with one least-square cubic spline per donor for visualization (the fitted splines serve only as visual aids and were not used for statistical analysis). Spectra are given for healthy young (<51 years, *purple*), healthy aged (>80 years with unaffected macula, *green*), and AMD (*yellow*). Spectra are shown for the fovea, perifovea, and near-periphery. Variations are apparent not only among the groups but also among individual donors.

When plotted by retinal location (Fig. 4B), foveal cells appear to have higher PC1 values (implying a flatter emission curve) compared to the perifovea and near-periphery. This is supported by the results of the Kruskal–Wallis test, which reject the null hypothesis of an identical median for all groups with *P* = 0.001.

Further subanalyses could not be performed due to preparative cell loss in advanced AMD cases.

### Associations Between Spectra and RPE Granule Load

The analysis revealed several significant associations between spectral emissions and abundance of granules. In the following, results are given in the format of the association coefficient (*au*) ± standard error and *P*.

For both healthy groups, we observed a negative association between PC1 (i.e., the flatness of the emission spectrum) and the number of lipofuscin granules (young: −0.178 ± 0.044, *P <* 0.001; aged: −0.458 ± 0.072, *P <* 0.001). In other words, cells with steeper spectra tended toward having more lipofuscin granules. This effect, however, was not present in AMD (0.046 ± 0.155, *P* = 0.769). For this reason, the association coefficients between the aged and AMD groups differ significantly (*P* = 0.003).

Between lipofuscin abundance and PC2 (i.e., the blue shift of the peak), there was a weak association in the healthy young group (young: 0.137 ± 0.063, *P* = 0.030). No such association was observed in the others (healthy aged: 0.054 ± 0.084, *P* = 0.518; AMD: −0.013 ± 0.096, *P* = 0.891). This indicates that a link between lipofuscin abundance and the peak blue shift, if it exists, is tenuous. No significant difference between the healthy aged and AMD coefficients was observed (*P* = 0.596).

For melanolipofuscin, a significant association with PC1 was observed in all three groups (healthy young: 0.106 ± 0.019, *P <* 0.001; healthy aged: 0.154 ± 0.028, *P <* 0.001; AMD: 0.651 ± 0.160, *P* = 0.003). The association strength for AMD was significantly higher (*P* = 0.002) which means that the abundance of melanolipofuscin appears to be associated with flatter emission spectra. At the same time, this effect is markedly more pronounced in AMD eyes.

Contrary to lipofuscin, melanolipofuscin abundance exhibited a weak negative association with PC2, although as before the effect size was small and either barely or not significant (healthy young: −0.07 ± 0.03, *P* = 0.018; healthy aged: 0.068 ± 0.034, *P* = 0.045; AMD: −0.223 ± 0.132, *P* = 0.094). We did not observe a significant difference between the association strengths for healthy aged and AMD (*P* = 0.255).

## Discussion

In this study, we examined spectral emission properties in over 5000 RPE cells from both healthy and AMD-affected human donor eyes at exactly predefined retinal locations. Our investigation characterized RPE spectral curves with increasing AF emission from 490 nm up to 570 to 580 nm. Crucially, we corroborated earlier studies that identified a spectral shift of peak emission toward shorter wavelengths in AMD-affected RPE cells—a shift most notable at the fovea and perifovea regions in this study. Moreover, linear mixed-effect modeling confirmed that the granule load for both lipofuscin and melanolipofuscin are intertwined with the shape of the emission spectrum. Interestingly, this relationship varied between AMD patients and healthy subjects. These findings may offer invaluable guidance for in vivo studies that aim to employ single-cell RPE imaging techniques.

### Autofluorescence Alterations in RPE AF During Aging

RPE cell bodies house the primary source of AF in the outer retina, encompassing organelles such as lipofuscin, melanolipofuscin, and melanosomes.^6^^,^^7^^,^^34^^,^^35^ Clinically, excitation with short-wavelength light showcases the signals from lipofuscin and melanolipofuscin in in vivo fundus AF.^34^^,^^36^

Existing studies indicate an increase in autofluorescence intensity during aging, especially in the first three decades,^37^^–^^40^ although it continues throughout life.^6^^,^^41^^,^^42^

However, merely relying on total AF intensity may be misleading, as it fails to consider other pertinent features such as granule types, numbers, and spectral data. In our single-cell approach, peak emission spectra were found to be 579 nm for fovea and perifovea, independent of age. Near-periphery RPE from healthy young donors had peak emissions at 575 nm and 579 nm. To summarize, unlike total AF that increases steadily during aging, the effect of age was miniscule on RPE spectra. Thus RPE spectral changes may be an accurate way of differentiating normal age-based changes from disease.

### Influence of Retinal Location and Tissue Preparation on RPE AF

Our study delved deeper into spectra from RPE cells at distinct retinal locations. Unlike previous investigations, our methodology was unique in its separate evaluation of retinal locations and its adoption of a single-cell en face imaging approach for RPE AF analysis.^5^^,^^13^^–^^15^^,^^21^^,^^43^ Various factors might account for the disparities observed in peak emissions among different studies, such as regional RPE autofluorescence variations, differing setups and excitation wavelengths, and tissue preparation. Marmorstein and colleagues^13^ found spectral peaks at 555 nm using whole macular flatmounts. Early studies reported RPE spectral peaks at 610 nm when excited at 510 nm for human postmortem flatmounts without signs of pathology (donors 60–72 years old). Feldman and colleagues^14^ found AF peaks of fresh-fixed suspended RPE cells at 575 nm for excitation at 488 nm, independent of age (similar as our results), and Schultz and colleagues^15^ found peak fluorescence at 610 nm in fixed and fresh frozen RPE cross-sections of healthy eyes from donors aged 80 years or older.^34^

Our results showed emission spectra at 575 to 580 nm dependent on retinal location. The impact of tissue preparation is a possible explanation; our study did not use suspended cells or cross-sections, but RPE flatmounts.^39^^,^^43^ These discrepancies within the studies demonstrate that absolute values of peak emission may vary by location, excitation wavelength, and tissue preparation. It is therefore pivotal to keep these factors constant when comparing health and disease.

### Observations in AMD-Affected RPE Cells

Alterations in fundus AF and granule distribution in AMD-affected eyes go beyond those seen during aging. Reduced overall AF levels in qAF imaging have been detected by independent groups.^10^^,^^11^^,^^44^^–^^46^ These clinical findings can be histologically confirmed, as AMD-affected RPE cells showed overall reduced AF through aggregation and degranulation.^3^^,^^47^ With AMD disease onset, proportionally reduced lipofuscin and increased melanolipofuscin were observed histologically when compared to healthy aged tissues.^3^^,^^9^

Marmorstein and colleagues^13^ investigated RPE AF and spectra in macular cross-sections and found no changes in spectral peaks and intensities between healthy and AMD tissues, whereas a slight hypsochromic spectral shift with increased AF intensity at the rising edge for AMD spectra was shown recently.^15^ Furthermore, an increase in AF intensities was detected for suspended AMD-affected RPE cells, but a shift of the spectral peak to shorter wavelengths in AMD tissues was detected only in two affected eyes.^16^^,^^48^^,^^49^

We also found a slight hypsochromic shift of the peak AF of RPE cells at the fovea in AMD eyes. Results for the perifovea were weaker, but suggestive, but no clear effects at the near-periphery were observed (AMD cells showed a hypsochromic shift compared to healthy aged cells, but healthy young cells peaked between the two). Extracellular deposits (soft drusen material in several forms and basal laminar deposits) in AMD-affected eyes are most frequent under the fovea.^50^^,^^51^ Our information on single-cell RPE spectra at defined retinal locations is thus important to differentiating between changes due to advanced age and damage caused by progressing AMD.^3^^,^^6^^,^^9^

The origin of the blue shift in RPE spectra remains unclear. Altered intracellular RPE granule accumulation could be indicative of the histologically identified proportionally low lipofuscin and high melanolipofuscin fraction, especially at the fovea, further enhanced in AMD.^9^^,^^44^ Our results show a link between lipofuscin content per cell and the shape of the emission spectrum curve. This supports the assumption that granule redistribution and/or loss in AMD-affected RPE cells leads to clinically visible AF alterations.

The observed blue shift was of a different extent in most studies presented, which can be explained by the following factors: response to different excitation wavelengths, different fluorophores in the RPE dependent on retinal location, changes in fluorophore composition, and distribution with age and disease. Studies on blue versus green AF demonstrate how tweaking the excitation wavelength by just 30 nm can result in altered autofluorescence response of RPE cells, albeit both originate from lipofuscin and melanolipofuscin.^52^ This might be related to several fluorophores being excited to a different extent (e.g., A2E has a maximum excitation at 430 nm).^53^ Given the impossibility of keeping all of these factors constant between studies, the extent of the blue shift varies, but the overall trend appears to be reproducible.

Mass spectrometry provides further evidence for different compositions of RPE granules.^54^^,^^55^ Lipofuscin granules in AMD-affected RPE cells contained higher levels of bisretinoid oxidation products,^16^ which might play a role in degenerative cell pathophysiology.^56^ However, one oxidized bisretinoid (monofuran-A2E) is much higher in peripheral retina than central retina, as are several known bisretinoids, raising questions about their role in macular diseases.^55^

### Strengths and Limitations

The strength of our study lies in the expansive data obtained from numerous RPE cells across defined locations, age groups, and in the context of AMD. However, certain challenges such as the biomechanical fragility of sub-RPE deposits, which may result in underrepresented foveal cells in AMD samples, are inherent limitations.^51^ Due to the loss of RPE cells, the sample size was not large enough to examine differences within the heterogenic spectrum of AMD. This may have led to an underestimation of spectral changes in AMD. The selected excitation wavelength and potential postmortem changes, along with cryoprotection considerations, could also introduce some biases.^13^^,^^14^^,^^57^^,^^58^ Finally, our method of using RPE flatmounts, although advantageous to rule out other retinal influences, disregards the impact of the apical processes of the RPE. Apical processes possess a high number of melanosomes that also contribute to the autofluorescent signal.

## Conclusions

In this extensive study, we employed a single-cell en face approach to scrutinize RPE AF spectra based on age, retinal location, and AMD presence. This has allowed, for the first time, to the best of our knowledge, the attribution of differences in AF properties to specific retinal areas. Furthermore, our insights into the relationship between the emission spectrum shape and lipofuscin content, especially in AMD, might prove pivotal for in vivo cellular-level AF imaging of the RPE in both healthy individuals and AMD patients.

## Supplementary Material

Supplement 1

Supplement 2

Supplement 3
