# Supplementary material for: Spectral Analysis of Human Retinal Pigment Epithelium Cells in Healthy and AMD Eyes
Source: Invest Ophthalmol Vis Sci. 2024 Jan 3;65(1):10. doi: 10.1167/iovs.65.1.10 (PMC10768704; doi:10.1167/iovs.65.1.10)
Supplement: Supplement 1 [file iovs-65-1-10_s001.pdf]

Bourauel, Vaisband et al.: Spectral Analysis of Human Retinal Pigment Epithelium Cells in Healthy and AMD Eyes. IOVS, 2023.

Supplementary Figure 1: Retinal locations for autofluorescence spectral imaging.

The spectral imaging was done at three predefinded locations. Based on the precise location of the fovea (see Methods in Ach et al.[1]), imaging was performed at the fovea, perifovea, and near-periphery. In some cases, predefined locations felt into areas where no RPE cells were available (i.e., due to preparation artifacts) and microscope stage was slightly moved around this area to capture enough RPE cells. Therefore, distances to fovea might slightly differ between tissues. OD: optic disc.

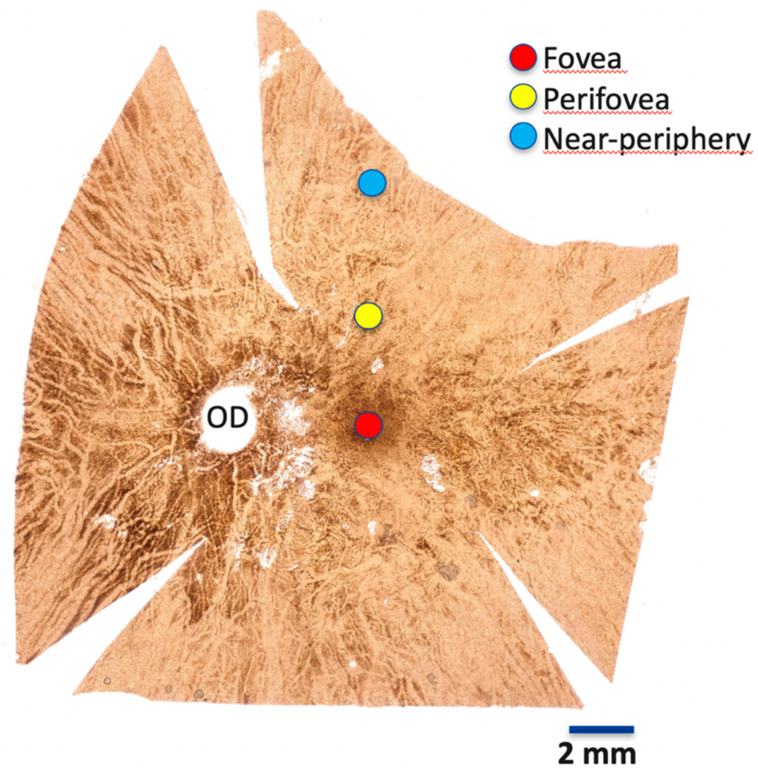

Reference:

[1] Ach T, Huisingh C, McGwin G, et al. Quantitative Autofluorescence and Cell Density Maps of the Human Retinal Pigment Epithelium. *Investigative Opthalmology & Visual Science*. 2014;55(8):4832. doi:10.1167/iovs.14-14802
