# Supplementary material for: Spectral Analysis of Human Retinal Pigment Epithelium Cells in Healthy and AMD Eyes
Source: Invest Ophthalmol Vis Sci. 2024 Jan 3;65(1):10. doi: 10.1167/iovs.65.1.10 (PMC10768704; doi:10.1167/iovs.65.1.10)

Bourauel, Vaisband et al.: Spectral Analysis of Human Retinal Pigment Epithelium Cells in Healthy and AMD Eyes. IOVS, 2023.

Supplementary Figure 2: Violin plots of median PC values in donor eyes across different localizations. The tendency of AMD eyes towards higher PC2 values (and thus a hypsochromic shift) is visible in all localizations, but is most pronounced and statistically significant at the fovea. The bandwidth for the violin plot kernel density estimation was determined according to the Scott rule.

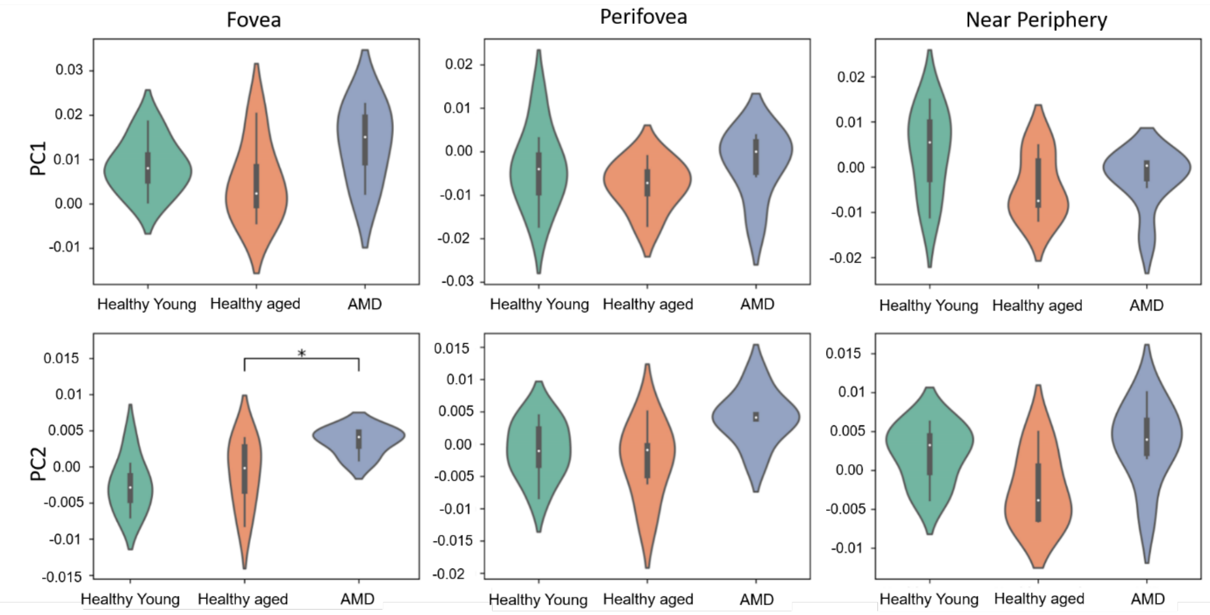

Supplement: Supplement 2 [file iovs-65-1-10_s002.pdf]
