# Supplementary material for: Spectral Analysis of Human Retinal Pigment Epithelium Cells in Healthy and AMD Eyes
Source: Invest Ophthalmol Vis Sci. 2024 Jan 3;65(1):10. doi: 10.1167/iovs.65.1.10 (PMC10768704; doi:10.1167/iovs.65.1.10)
Supplement: Supplement 3 [file iovs-65-1-10_s003.pdf]

Bourauel, Vaisband et al.: Spectral Analysis of Human Retinal Pigment Epithelium Cells in Healthy and AMD Eyes. IOVS, 2023.

Supplemental Table 1: FIJI Plugins for spectral analysis.

**Task**	**Used FIJI Plugin**
Generation of single spectra per cell	Create spectrum jru v1
Combine all spectra per tissue and location for average spectrum	Combine all trajectories jru v1
Integral normalization of spectra for comparability	Normalize trajectories jru v1
